# Biomarkers identification by a combined clinical and metabonomics analysis in Henoch-Schonlein purpura nephritis children

**DOI:** 10.18632/oncotarget.23207

**Published:** 2017-11-24

**Authors:** Lin Sun, Biao Xie, Qiuju Zhang, Yupeng Wang, Xinyu Wang, Bing Gao, Meina Liu, Maoqing Wang

**Affiliations:** ^1^ Department of Epidemiology and Biostatistics, Public Health College, Harbin Medical University, Harbin, P. R. China; ^2^ Department of Nutrition and Food Hygiene, Public Health College, Harbin Medical University, Harbin, P. R. China

**Keywords:** HSP, HSPN, biomarkers, metabonomics, clinical

## Abstract

**Background:**

In children with Henoch-Schonlein purpura (HSP), the severity of Henoch-Schonlein purpura nephritis (HSPN) is considered responsible for the prognosis of HSP. The pathological process from HSP to HSPN is not clear yet and current diagnostic tools have shortcomings in accurate diagnosis of HSPN. This study aims to assess clinical characteristics of HSP and HSPN, to identify metabolic perturbations involved in HSP progress, and to combine metabolic biomarkers and clinical features into a better prediction for HSPN.

**Methods:**

A total of 162 children were recruited, including 109 HSP patients and 53 healthy children (HC). The clinical characteristics were compared between HSPN and HSP without nephritis (HSPWN). The serum metabonomics analysis was performed to determine the metabolic differences in HSP and HC.

**Results:**

Among 109 HSP children, 57 progressed to HSPN. The increased D-dimer level was significantly associated with renal damage in HSP. The metabonomic profiles revealed alterations between various subgroups of HSP and HC, making it possible to investigate small-molecule metabolites related to the pathological process of HSP. In total, we identified 9 biomarkers for HSP vs. HC, 7 for HSPWN vs. HC, 9 for HSPN vs. HC, and 3 for HSPN vs. HSPWN.

**Conclusions:**

(S)-3-hydroxyisobutyric acid, p-Cresol sulfate, and 3-carboxy-4-methyl-5-pentyl-2-furanpropanoic acid were found associated with the progress of HSP to HSPN. Moreover, resulting biomarkers, when combined with D-dimer, allowed improving the HSPN prediction with high sensitivity (94.7%) and specificity (80.8%). Together these findings highlighted the strength of the combination of metabonomics and clinical analysis in the research of HSP.

## INTRODUCTION

Henoch-Schonlein purpura (HSP) is a systemic small-vessel vasculitis characterized by tetrad manifestations: palpable skin purpura, arthralgia/arthritis, intestinal symptoms, and kidney damage [1-3]. Renal HSP, known as Henoch-Schonlein purpura nephritis (HSPN), is the most common and severe complication, and a major factor affecting the long-term outcome of patients. An early and accurate diagnosis of HSPN is important in HSP prognosis and early personalized medicine. Renal biopsy and routine urinanalysis (UA) tests are two major methods available for detecting HSPN, however, both of which were unsatisfactory in practical clinic cases. Renal biopsy is not acceptable for pediatric patients because of its invasiveness, which may lead to hemorrhagic injury of the urinary system; moreover, renal biopsy reveals kidney damage when nephrotic losses have already occurred, which is inadequately predicted HSPN before nephritis strikes. UA tests fall short in sensitivity and specificity. Previous studies have reported that HSP patients with normal UA also exhibit various degrees of renal pathological lesion in the renal biopsy [3]. Slight differences in clinical features between HSPN and HSP without nephritis in the onset of the complaint account for the challenge in the diagnosis of HSPN. Yet, subtle alterations in metabolic pathways related to renal involvement in HSPN patients have already proceeded preceding the abnormal changes of urine analysis or renal biopsy. Given this background, we considered metabonomics [4] as an appropriate tool for determining underlying variation of metabolites and provide novel insight into the metabolism status of affected patients. Additionally, some clinical characteristics related to HSP with renal damage, which can serve as risk factors for the occurrence of HSPN, may provide a supplement for the prediction of renal lesion in HSP patients.

We conducted our study from two aspects: first, we profiled the clinical characteristics in order to investigate the clinical features related to the renal involvement in children with HSP; second, we performed a serum metabonomics analysis using the ultra performance liquid chromatography quadrupole time-of-flight tandem mass spectrometry (UPLC-Q-TOF-MS/MS), aimed at searching the slight metabolic changes in the progression of HSP and identifying specific biomarkers for children with HSPN. The combination of clinical features and metabolic biomarkers might facilitate clinical diagnosis for renal involvement in HSP in initial stages.

## RESULTS

### Characteristics of patients

**Figure 1:**
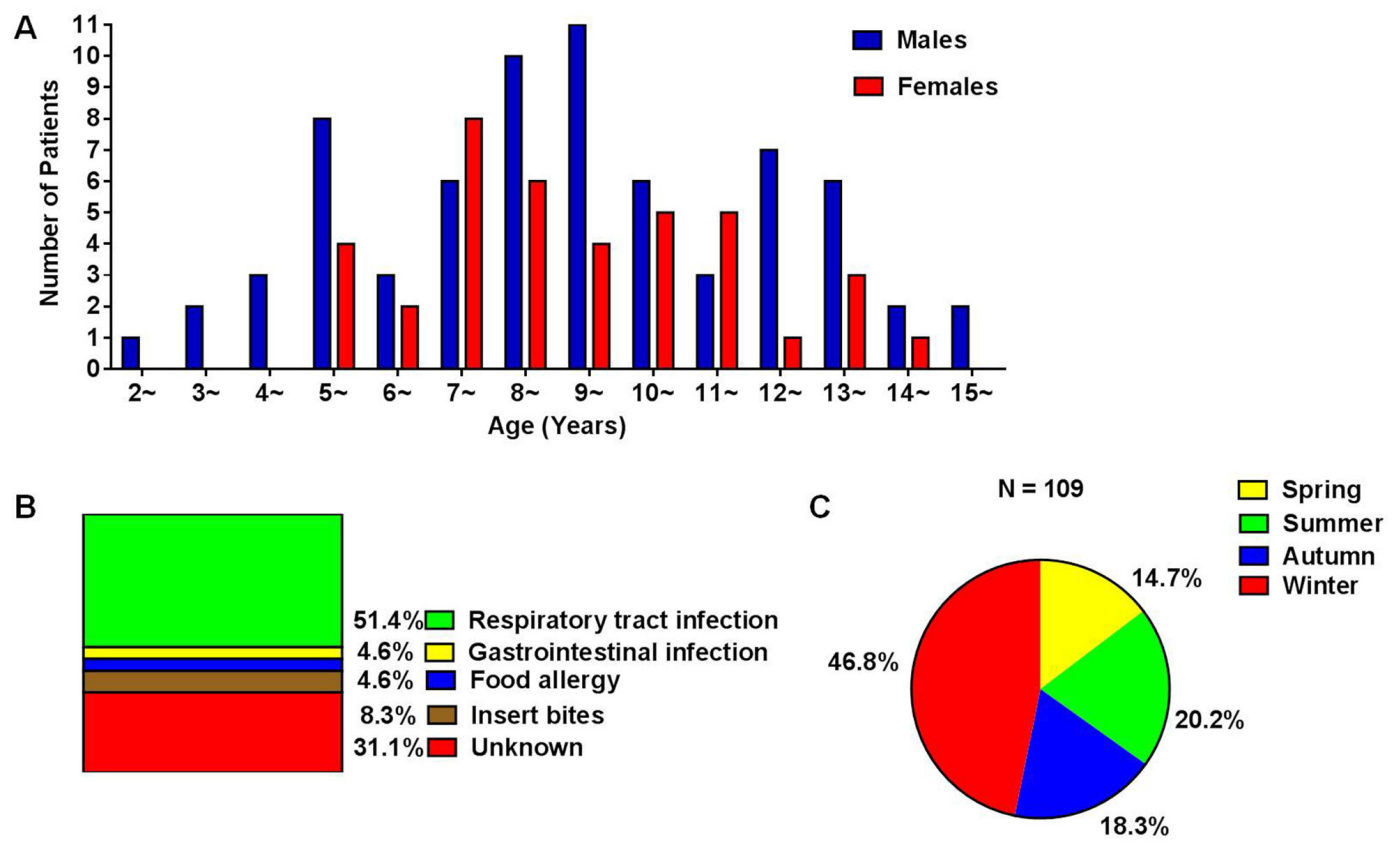
**(A)** Age and gender distribution of HSP children; **(B)** predisposing factors for HSP; **(C)** seasonal distribution of HSP onset.

A total of 109 children with HSP were enrolled in the study, including 70 males and 39 females. The ratio of male to female was 1.79:1. The average age of onset of HSP was 8.74 ± 2.99 years (range of 2 to 15 years). About 76.1% cases occurred in those aged 5-13 years, with male to female ratio of 1.58:1 (Figure 1A). The peak season for HSP attack was winter, while HSP onset occurred during all seasons (Figure 1B). Most cases had a certain predisposing factor before disease onset, among which 56 had a respiratory tract infection, 5 had a gastrointestinal infection, 5 had eaten special food like egg and fish that caused a food allergy, and 9 had been accepted a vaccine injection, while 34 cases were triggered by unknown factors (Figure 1C). A total of 53 healthy children were enrolled, including 28 males and 25 females. The ratio of male to female was 1.12:1. The average age of 53 healthy children was 7.91 ± 3.07 (range of 2 to 14 years). There were no significant differences in gender or age between HSP and HC.

The main clinical features of 109 children with HSP were summarized in Table 1. All cases appeared skin rash. 56% patients affected gastrointestinal symptoms including abdominal pain, gastrointestinal bleeding, diarrhea and vomiting. 48% children developed joint symptoms including arthralgia and arthritis. 52% cases had signs of renal damage, among which 9% appeared isolated hematuria, 10% appeared isolated proteinuria, and 33% appeared hematuria except proteinuria.

**Table 1:** Clinical features of HSP children

Clinical features	N	Percent
skin purpura	109	100
digestive symptoms	58	53
Joint involvement	48	44
renal involvement	57	52
isolated hematuria	10	9
isolated proteinuria	11	10
hematuria and proteinuria	36	33

Clinical characteristics were compared in HSPWN and HSPN (Table 2). There were no significant differences in gender distribution, age, the incidence of joint involvement and abdominal pain, white blood count, the percentage of neutrophils, platelet count, mean platelet volume or cystatin C level between HSPWN and HSPN. Meanwhile, there were significant difference in the occurrence of bloody stools and positive occult blood in stool between HSPN and HSPWN, which indicated that gastrointestinal bleeding occurred more frequently in children with renal damage. Additionally, the increase of C-reactive protein and D-dimer was seen more often in HSPN than in those HSPWN, with a P value of 0.022 and 0.000 respectively. The result of multivariate logistic regression was shown in Table 3. Raised D-dimer level was the only independent risk factor related to renal involvement in children with HSP.

**Table 2:** Clinical and laboratory data at disease onset in children with HSP with or without renal involvement

Parameter	HSPWN n = 52	HSPN n = 57	P value
Gender			0.297
Male	36 (69%)	24 (60%)	
Female	16 (31%)	22 (40%)	
Age, years	8.58 ± 2.64	8.91 ± 2.89	0.548
Arthralgia or arthritis	21 (40%)	27 (47%)	0.463
Abdominal pain	23 (44%)	24 (42%)	0.823
Bloody stools	11 (21%)	22 (39%)	0.048
Occult blood in stool	16 (31%)	29 (51%)	0.033
White blood cell count, ×10^9^/l	8.98 ± 3.53	9.63 ± 4.70	0.414
Neutrophils, %	56.14 ± 18.29	59.20 ± 17.40	0.373
Platelet count, ×10^9^/l	329.08 ± 92.94	357.14 ± 100.87	0.135
Mean platelet volume, fl	10.03 ± 0.75	10.10 ± 0.74	0.620
Cystain C, mg/l	0.88 ± 0.39	0.95 ± 0.48	0.367
C-reactive protein increase	12 (23%)	25 (44%)	0.022
D-dimer increase	8 (15%)	38 (67%)	0.000

**Table 3:** Risk factors for the renal damage in HSP in children

Risk factor	B	P value	OR	95% CI
Bloody stools	0.126	0.867	1.134	0.259 - 4.966
Occult blood in stool	0.383	0.585	1.467	0.370 - 5.811
C-reactive protein increase	0.941	0.058	2.564	0.967 - 6.794
D-dimer increase	2.349	0.000	10.473	3.977- 27.576

### Metabolic profiles of HSP patients and healthy children

As displayed by PCA scores plot of all experiment injections (see Supplementary Figure 1), the QC samples were tightly clustered between HC and affected children. The %RSD of all metabolites from QC samples varied from 1.36 to 84.83% with a median of 20.88%, further, the %RSD of 9 selected metabolites varied from 1.78 to 19.89% with a median of 8.22% (see Supplementary Figure 2). These results indicated a generally robust operating condition of UPLC-Q-TOF-MS/MS instruments.

The unsupervised PCA detected obvious separations in HSPN vs. HC and HSPWN vs. HC (see Supplementary Figure 3). The results suggested that HSP accounted for the distinct metabolic disturbances in serum of affected children. Yet, the PCA score plot indicated rare tendency of differences in metabonomics profiling for HSPN vs. HSPWN.

To investigate these disturbances and develop specific biomarkers for HSP, multivariate classification modeling using PLS-DA coupled with feature selection was used to differentiate HSP and HC groups. Clear separation was obtained: 3 components with cumulative R2Y at 0.959 and Q2 at 0.929, see Figure 3A.

**Figure 2:**
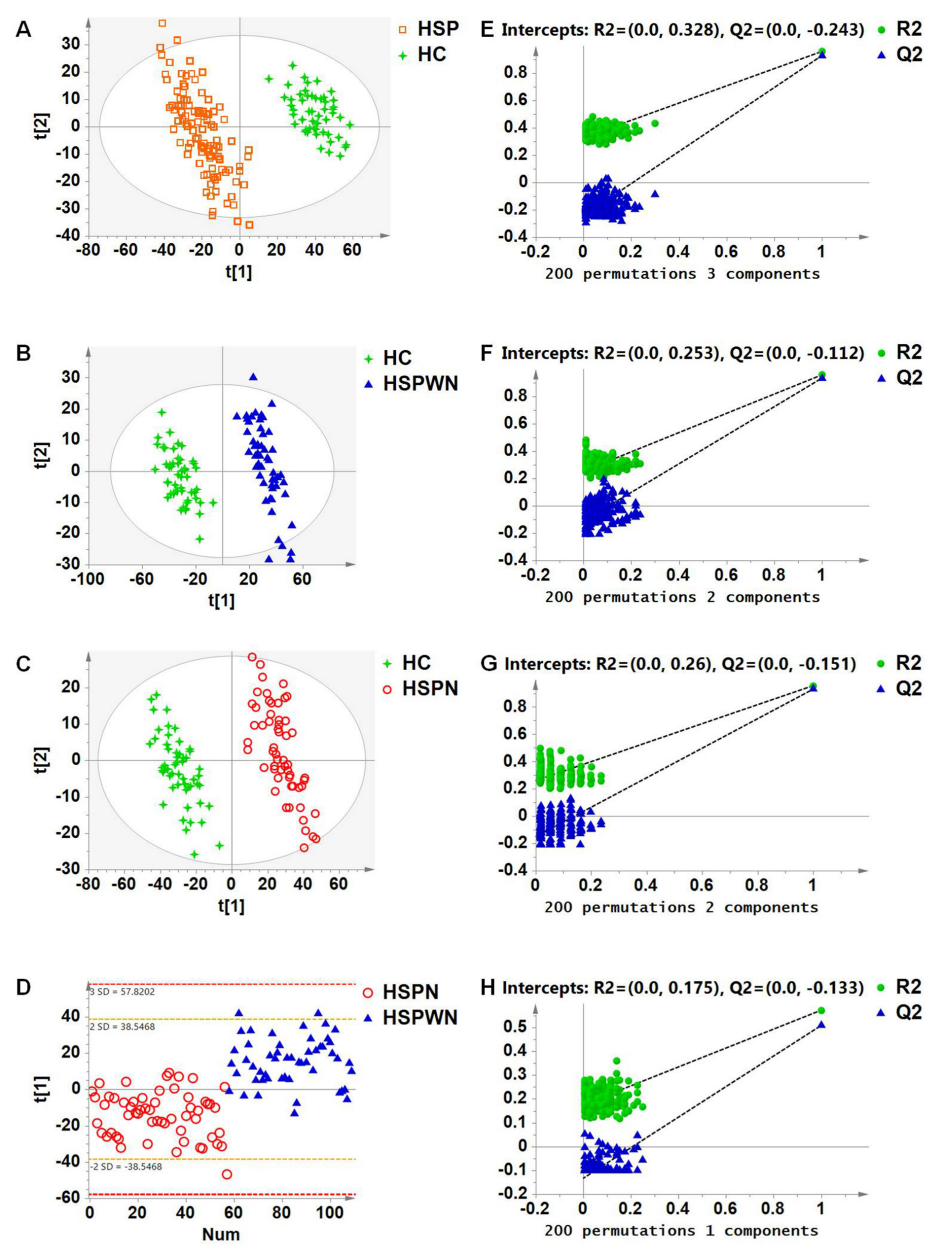
**(A)** PLS-DA score plot for HSP vs. HC; **(B)** PLS-DA score plot for HSPWN vs. HC; **(C)** PLS-DA score plot for HSPN vs. HC; **(D)** PLS-DA score plot for HSPN vs. HSPWN; **(E)** validation plot for HSP vs. HC; **(F)** validation plot for HSPWN vs. HC; **(G)** validation plot for HSPN vs. HC; **(H)** validation plot for HSP vs. HSPWN.

**Figure 3:**
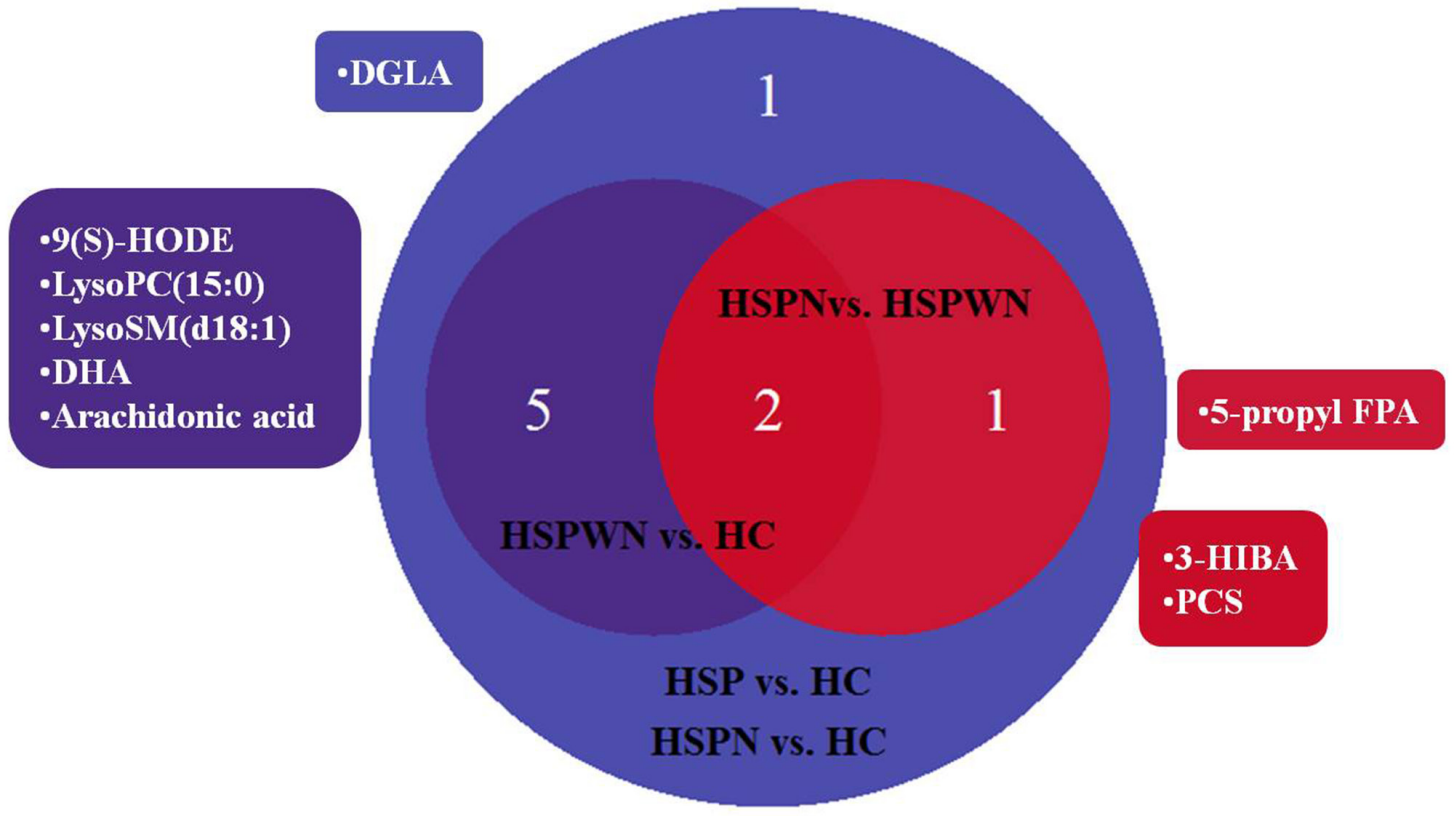
Venn plot for all biomarkers in two-two comparison among three groups

To discover pathological alterations in the progression of renal involvement in HSP and detect specific biomarkers for HSPN, we compared the various subgroups of HSP (HSPWN and HSPN) to HC and with each other using PLS-DA. Clear differences were obtained for the following: HSPWN vs. HC: 2 components with cumulative R2Y at 0.958 and Q2 at 0.934; HSPN vs. HC, 2 components with cumulative R2Y at 0.956 and Q2 at 0.930; HSPN vs. HSPWN, 1 component with cumulative R2Y at 0.572 and Q2 at 0.508, see Figure 2B, 2C and 2D respectively.

The validation plots generated from 200 permutation tests and the results of the CV-ANOVA test strongly manifested the validity of four classification models, see Figure 2E-2H and Supplementary Tables 1-4 respectively.

### Identification of biomarkers

By 4 comprehensive cross-comparisons of different groups, a total of 9 differential metabolites were identified, from which 7 were confirmed by the MSMS spectra from web databases or MassFragment application manager, and 2 was identified by searching the web database (for detailed information, see Supplementary Data).

Following VIP values of PLS-DA models with a threshold of 1.0 and P values of independent sample *t-tests* with a threshold of 0.05, there were 9 metabolic biomarkers for HSP vs. HC, 7 for HSPWN vs. HC, 9 for HSPN vs. HC, and 3 for HSPN vs. HSPWN, as summarized in Table 4. The Venn plot depicted the overlap of biomarkers for cross-comparisons, as shown in Figure 3.

**Table 4:** Serum metabolic biomarkers for 4 cross-comparisons

m/z	RT	Identity	HSP vs. HC			HSPWN vs. HC			HSPN vs. HC			HSPN vs. HSPWN		
	(min)		VIP	P-value	FC^1^	VIP	P-value	FC^2^	VIP	P-value	FC^3^	VIP	P-value	FC^4^
103.039	1.74	3-HIBA	1.53	0.000	4.65	1.27	0.000	3.76	1.87	0.000	5.47	1.50	0.000	2.09
187.004	2.90	PCS	2.49	0.000	1.81	1.71	0.004	1.57	3.54	0.000	2.04	3.28	0.001	1.48
267.124	3.89	5-propyl FPA	1.72	0.000	1.76	0.62^*^	0.030	1.25	2.56	0.000	2.22	4.04	0.000	1.77
295.229	4.69	9(S)-HODE	1.52	0.000	-6.56	1.37	0.000	-7.20	1.50	0.000	-6.06	0.19^*^	0.304^*^	1.19
480.310	6.04	LysoPC(15:0)	1.07	0.000	-2.38	1.08	0.000	-2.86	1.01	0.000	-2.07	0.69^*^	0.002	1.38
464.316	6.97	LysoSM(d18:1)	2.47	0.000	-9.89	2.27	0.000	-13.24	2.44	0.000	-8.04	0.66^*^	0.006	1.65
327.232	8.07	DHA	1.62	0.000	-1.43	1.57	0.000	-3.06	1.54	0.000	-3.65	0.62^*^	0.318^*^	-1.19
303.232	8.59	Arachidonic acid	4.52	0.000	-2.68	4.14	0.000	-2.73	1.06	0.000	-2.63	0.30^*^	0.667^*^	1.04
305.249	9.58	DGLA	1.04	0.000	-1.26	0.97^*^	0.000	-1.27	1.50	0.000	-1.25	0.05^*^	0.869^*^	1.01

^1^FC with a positive value suggests that the concentration of a certain metabolite is up-regulated in HSP compared to HC.

^2^FC with a positive value suggests that the concentration of a certain metabolite is up-regulated in HSPWN compared to HC.

^3^FC with a positive value suggests that the concentration of a certain metabolite is up-regulated in HSPN compared to HC.

^4^FC with a positive value suggests that the concentration of a certain metabolite is up-regulated in HSPN compared to HSPWN.

^*^VIP < 1.0 or P > 0.05.

**Figure 4:**
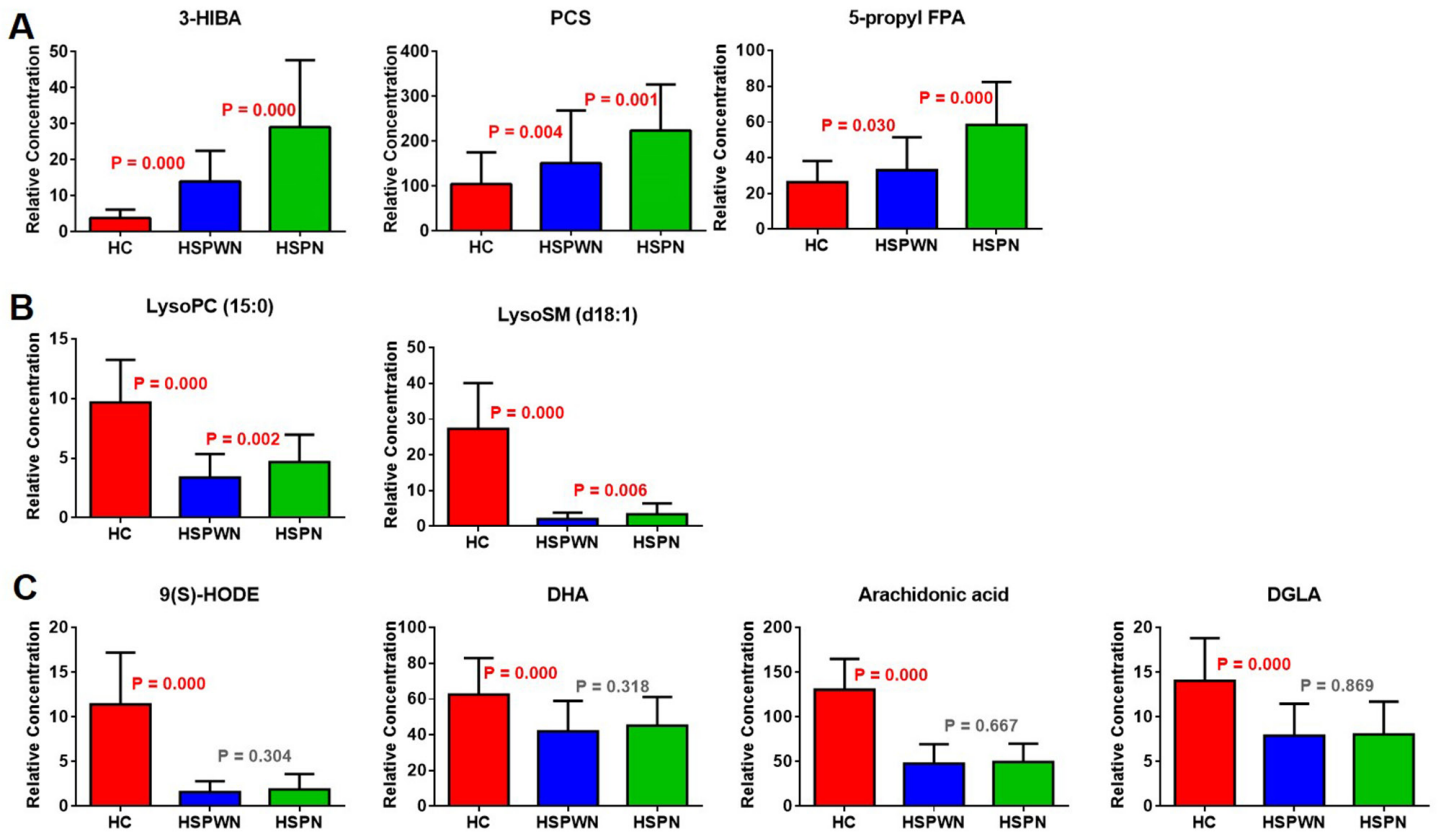
Changing patterns of differential metabolites from HC group across HSPWN and HSPN groups: **(A)** Type A biomarkers; **(B)** Type B biomarkers; **(C)** Type C biomarkers.

Biomarkers were divided into 3 types according to their various tendencies in the process of HSPN, as shown in Figure 4. 5 metabolites were significantly perturbed in HSPWN vs. HC and HSPN vs. HSPWN, among them, 3 continuously up-regulated in HSPWN compared to HC and HSPN compared to HSPWN were grouped into type A, comprising (S)-3-Hydroxyisobutyric acid (3-HIBA), p-Cresol sulfate (PCS), and 3-carboxy-4-methyl-5-pentyl-2-furanpropanoic acid (5-propyl FPA); the other 2 showing opposite change directions of concentration in HSPWN compared to HC and HSPN compared to HSPWN were grouped into type B, comprising LysoPC(15:0) and LysoSM(d18:1). 4 metabolite down-regulated in HSP compared to HC while undifferentiated expressed between HSPWN and HSPN were grouped into type C, comprising alpha-dimorphecolic acid (9(S)-HODE), 8,11,14-Eicosatrienoic acid (DGLA), docosahexaenoic acid (DHA), and arachidonic acid.

**Figure 5:**
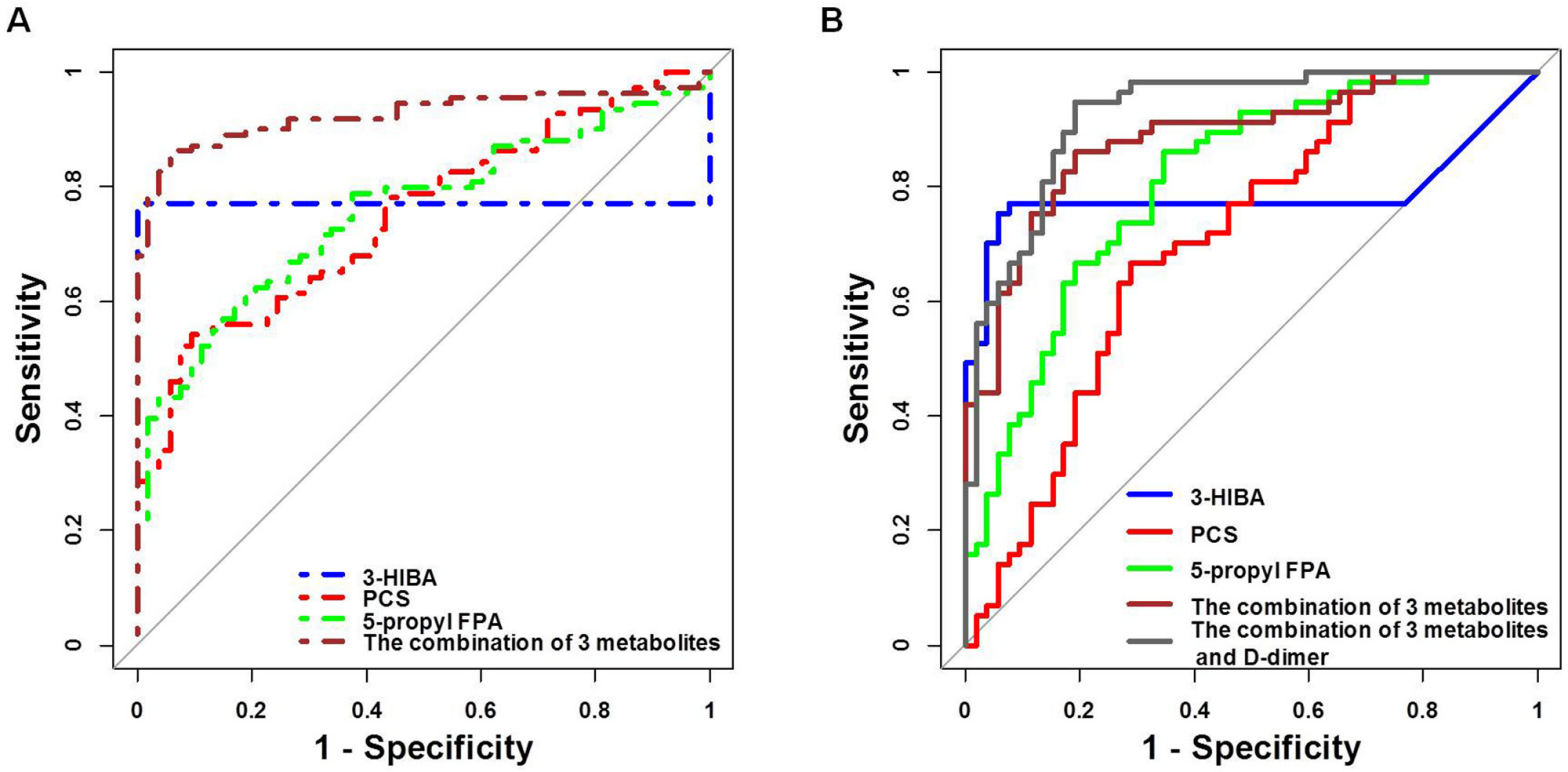
**(A)** ROC curves of biomarkers for HSP prediction; **(B)** ROC curves of biomarkers for HSPN prediction.

The ROC presentations for HSP vs. HC and HSPN vs. HSPWN appear in Table 5 in the Supplement. The prediction of HSP and HSPN in children was robust, where the area under the curve (AUC) of 9 biomarkers for HSP vs. HC and 3 biomarkers for HSPN vs. HSPWN ranged from 0.703 to 0.998 and 0.705 to 0.808, respectively. These results revealed that metabolic profiling could offer a highly accurate diagnosis of HSP and HSPN in children. The combination of 3-HIBA, PCS and 5-propyl FPA performed by logistic regression showed better discrimination, with AUCs of 0.918 for HSP vs. HC and 0.884 for HSPN vs. HSPWN (Table 5 and Figure 5).

**Table 5:** Results of AUCs for biomarkers

Biomarker	HSP vs. HC		HSPN vs. HSPWN	
	AUC	P-value	AUC	P-value
3-HIBA	0.771	0.000	0.786	0.000
PCS	0.750	0.000	0.705	0.000
5-propyl FPA	0.757	0.000	0.808	0.000
9(S)-HODE	0.992	0.000	-	-
LysoPC(15:0)	0.911	0.000	-	-
LysoSM(d18:1)	0.998	0.000	-	-
DHA	0.770	0.000	-	-
Arachidonic acid	0.982	0.000	-	-
DGLA	0.851	0.000	-	-
The combination of top biomarkers	0.925	0.000	0.884	0.000
The combination of top biomarkers and D-dimer	-	-	0.926	0.000

### Data integration to improve prediction

Metabonomics data were integrated with clinical data to improve the prediction for HSPN in children. Results of multivariate logistic regression analyses on 3-HIBA, PCS, 5-propyl FPA and D-dimer are shown in Table 6. All features were significant after multivariate adjustment. The combination of 3-HIBA, PCS, 5-propyl FPA and D-dimer strengthened the prediction capacity for HSPN, with the AUC of 0.926 (Table 5 and Figure 5).

**Table 6:** Logistic regression analysis of combined metabolic biomarkers and clinical risk factor

Parameter	B	P value	OR	95% CI
3-HIBA	0.043	0.024	1.043	1.006 - 1.083
PCS	0.007	0.008	1.007	1.002 - 1.012
5-propyl FPA	0.017	0.000	1.071	1.036 - 1.108
D-dimer increase	0.703	0.000	15.935	4.018- 63.186

## DISCUSSION

HSP is the most common small vessel vasculitis in the childhood, with an incidence of 6 to 24 per 100,000 children below 17 years old. HSPN is the most common and severe complication of HSP, which primarily accounts for the chronic consequences of HSP cases. The pathogenesis leading to HSP and HSPN are poorly investigated. Currently, the clinic has some limits in identifying HSPN. UA tests and renal biopsy are commonly used for diagnosis of HSPN with the deficiency in sensitivity, which lead to misdiagnosis of nephritis in some HSP cases. From the viewpoint of precisely identifying HSPN, it’s a pressing need to find biomarkers that more sensitively reflect the disorders of renal function. Metabonomics, capturing the underlying metabolic changes that reflect the body’s response to surrounding pathological stimulus, was considered as a suitable platform for specific characterization of HSPN.

In the present study, a total of 109 HSP children with or without renal involvement and 53 healthy children were enrolled. HSP occurs most frequently at age of 5 to 13 (76.1%), with the mean age of 8.74 ± 2.99 years. 64% of the affected children were boys and 36% were boys. The ratio of M to F was 1.79:1, which indicated HSP occurred more common in boys. This kind of gender related difference in HSP incidence was also reported in other studies. According to our data, HSP onset occurred around all seasons, with the highest incidence in winter. And about half of the cases (51.4%) had experienced a respiratory tract infection before HSP affecting. These might be due to the bad air quality and the obvious variety range of temperature changes in winter that made children tend to respiratory infections. The clinical analysis of systemic symptoms revealed significant differences in the occurrence of bloody stools and positive occult blood in stool, and the increase of C-reaction protein and D-dimer between HSPN and HSPWN according to univariate analysis. However, multivariate analysis showed that the raised D-dimer level was the only valid risk factor for renal involvement in the progress of HSP.

Metabolic profiling revealed significant metabolic differences among HC, HSPWN and HSPN groups. The PCA score plot of all samples displayed clear separation for HSP vs. HC, suggesting that HSP led to a series of metabolic changes in affected children. The PLS-DA model for HSP vs. HC revealed significant alterations of HSP progress. Ulteriorly, PLS-DA models for HSPWN vs. HC, HSPN vs. HC, and HSPN vs. HSPWN suggested some characteristic underlying pathophysiological changes associated with renal involvement in HSP. A total of 9 potential biomarkers were identified; with 9 significantly varied for HSP vs. HC, 3 significantly varied for HSPN vs. HSPWN, 7 significantly varied for HSPWN vs. HC, and 9 significantly varied for HSPN vs. HC.

Compared with healthy children, children with HSP had down-regulated 9(S)-HODE, DGLA, and DHA. 9(S)-HODE promotes the expression of plasminogen activator inhibitor type-1 (PAI-1) by activating peroxisomal proliferator-activated receptor- γ (PPAR-γ) in human endothelial cells. PAI-1 exerts great effect in inhibition of fibrinolysis. A low concentration of 9(S)-HODE hampered PPAR- **γ** activation and thus inhibited PAI-1 expression, resulting in active fibrinolysis, which caused mucocutaneous hemorrhage, manifested as skin purpura [6-8]. DGLA is the elongation of gamma-linolenic acid(GLA) and can be transformed toprostaglandin E1(PGE1). PGE1 is an inhibitor of platelet aggregation [9-11], making a great effect at vasodilatation. The down-regulation of DGLA reflected the decreased serum PGE1, might facilitate platelet aggregation and thrombosis, implying the coagulation disorders might be involved in the pathogenesis of HSP. DHA is metabolic product of DPA. Studies have demonstrated that the defect of DPA can lead to renal injury, skin damage, and impairment of connective tissue [12]. The appearance of skin purpura and articulus pain might be associated with decreased levels of DPA in HSP patients.

Compared with affected children without nephritis, children with HSPN up-regulated PCS, 5-propyl FPA, and 3-HIBA. PCS was a kind of protein-bound uremic toxins. The elevation of PCS could facilitate the prediction of the severity of renal impairment in chronic kidney disease (CKD) [13-14]^.^ 5-propyl FPA inhibited active tubular secretion and might serve as a indicator of tubular dysfunction. In addition, 5-propyl FPA accumulated and caused restrained active transports of organic acids in kidney. Previous studies have found a plasma 5-propyl FPA accumulation in patients with chronic renal damage [15-16]. 3-HIBA is an intermediate of L-valine metabolism. Valine is a branched-chain amino acid associated with renal impairment. The previous study observed a significantly reduced level of valine in arterial blood of chronic renal failure patients and inferred that valine levels directly made effect on GFR. Additionally, 3-HIBA was proven to inhibit key enzymes of energy metabolism in rats, including cytosolic and mitochondrial creatine kinase. Raised 3-HIBA levels might contribute to the renal lesion by causing mitochondrial dysfunction [17-19].

Biomarkers were divided into three groups according to various change trends. Biomarkers of type A and type B showed significant alterations in HC vs. HSPWN vs. HSPN, among which, type A had identical change directions in concentration between the progression from HSP to HC. Biomarkers of type C showed significant differences in HSPWN compared to HC but no differences between HSPN and HSPWN. The main aim of the present work was to investigate the progress of renal involvement in HSP and discover specific biomarkers for HSPN diagnosis, thus, we put more concern on the predictive abilities of type A metabolites. The combination of 3 kinds of type A biomarkers enhanced the discrimination ability, with AUCs of 0.925 for HSP vs. HC and 0.884 for HSPN vs. HSPWN. As a result, the combination of PCS, 5-propyl FPA and 3-HIBA was selected as top biomarkers for HSPN diagnosis. The combination of these top biomarkers and the clinical risk factor for renal involvement in HSP even performed better in the prediction of HSPN (AUC = 0.926, sensitivity = 94.7%, specificity = 80.8%).

Type B metabolites are unsatisfactory biomarkers for HSPN diagnosis due to their various change directions of concentration among HC, HSPWN and HSPN groups. However, this kind of metabolites is more valuable in investigating the onset of renal damage in HSP progress.

Type C metabolites might serve as biomarkers for differential diagnosis of HSPN and IgAN. HSPN and IgA nephropathy (IgAN) are deemed to share common pathogenesis, both of which characterized by IgA immune complex deposits. HSPN usually develops concomitant with or after the presence of palpable purpura which tends to an acute nature, while IgAN is a renal-limited disease with a relatively chronic course which tends to result in CKD [20-22]. On account of different clinical and pathological characteristics, clinical strategies and therapies generally differ for the two diseases. However, some HSPN children whose renal damage preceded skin rash were diagnosed with IgAN and applied therapy targeted on renal injury, resulting in delayed diagnosis and treatment [23]. Type C biomarkers, showing significant differences in HSPWN compared to HC but no difference between HSPN and HSPWN, might be used for discriminating HSPN from those IgAN without skin rash.

The present study has some limitations, in which no external sample set used for validation, no external sample set used for validation, and uncertain causal relationships between the biomarkers and the occurrence of HSP or HSPN. To the best of our knowledge, this is the first human study investigating not only the clinical features but also metabolic changes of HSP and HSPN using an UPLC-Q-TOF-MS/MS based metabonomics approach. Future study will put focus on the combination of clinic characteristics and metabolic features, leading to more completely understandings of HSP. More researches directed to biological interpretation are expected: which biomarkers are related to the severity of renal lesion in HSP, which dysregulations of pathways are involved in the onset and progression of HSP, or if any different metabolic changes occur between pediatric patients and adult patients.

In conclusion, the clinical analysis revealed that the increased D-dimer level was the independent risk factor for renal involvement in HSP, furthermore, the serum metabonomics analysis yielded novel insights into small-molecule metabolic alterations in the progression from HSP to HSPN. PCS, 5-propyl FPA and 3-HIBA, continuously varying across the progression of HSP, could serve as top biomarkers for HSPN. These metabolites, when combined with D-dimer, allowed improving the renal involvement prediction in children with HSP, which could be applied as an additional diagnostic tool for detecting HSPN in practical clinic cases.

## MATERIALS AND METHODS

**Figure 6:**
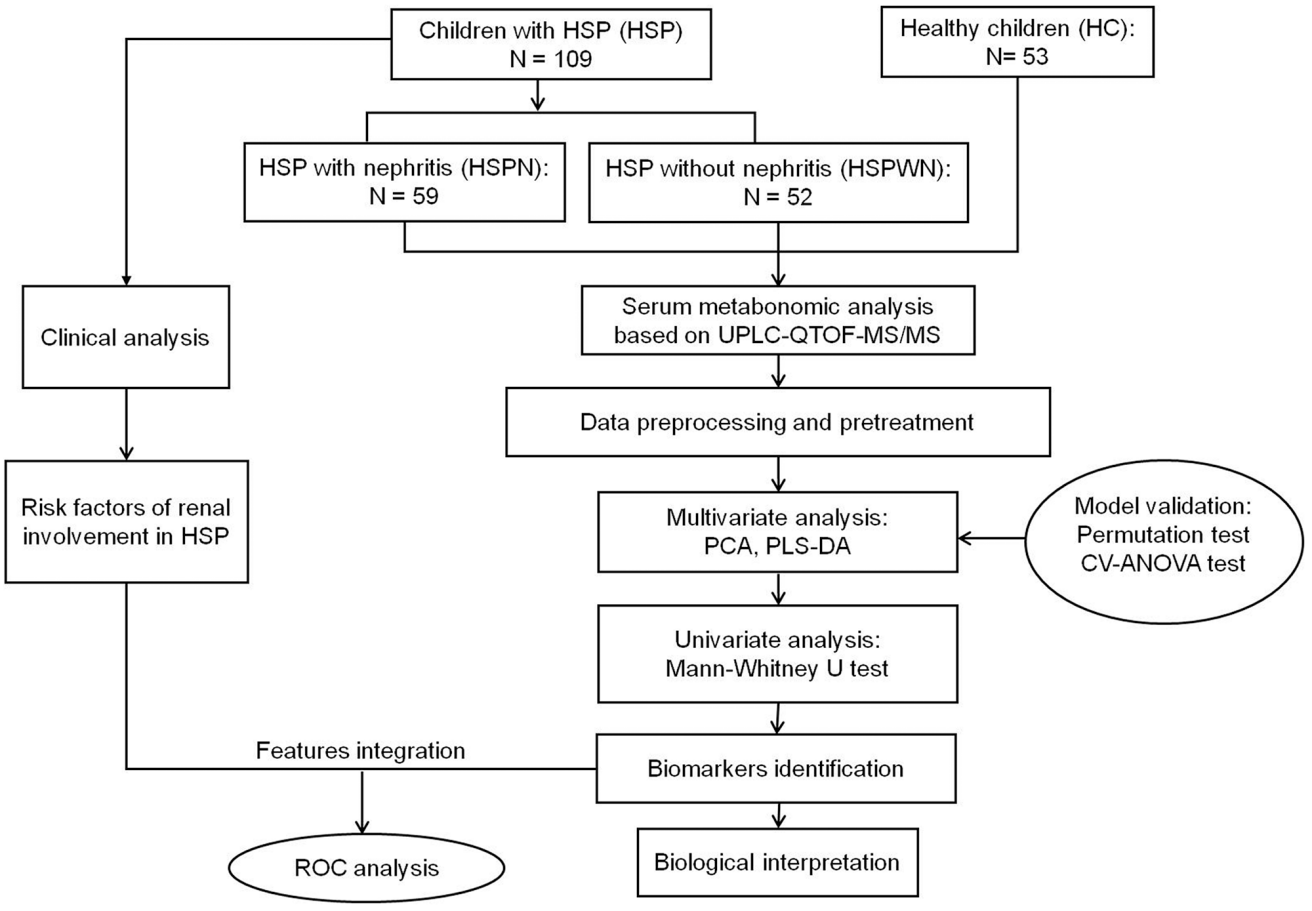
An overview of workflow utilized in serum metabonomics analysis of HSP

The overall workflow of serum metabonomics analysis utilized in this study is summarized in Figure 6.

### Subjects

The study protocol was approved by the Ethics Committee of Harbin Medical University. Informed consent forms were signed from all subjects before entering this project. Each participant was inquired to obtain a questionnaire and provide morning urination.

HSP was diagnosed according to the criteria defined by the European League against Rheumatism and Paediatric Rheumatology European Society [5]. HSPN was diagnosed as the existence of renal involvement during the first 6 months course of HSP, manifesting as hematuria and/or proteinuria. Hematuria is defined as gross hematuria or more than 5 red blood cells in high power field of microscopy. Patients presenting positive urine protein continuously three times during a week and those presenting negative urine protein but excess excretion of urine microalbumin were both considered as proteinuria. Children with regular treatment for a chronic disease and those with urinary tract infections were excluded from our study. All affected children were enrolled from the pediatric inpatient department of the Second Affiliated Hospital of Harbin Medical University from February 2013 to January 2014. The healthy controls were made up of healthy volunteers with the same age from the health examination center of the Second Affiliated Hospital of Harbin Medical University from February to March, 2013. At last, 109 children affected HSP (HSP group) and 53 healthy children (HC group) were enrolled for serum metabonomics analysis. Among 109 HSP children, 57 children developed renal involvement during the 3rd week of the follow-up period (HSP with nephritis, HSPN group), while another 52 children had no abnormal change in kidneys (HSP without nephritis, HSPWN group).

### Clinical analysis

The epidemiological features like age of disease onset and sex were collected. Also, clinical symptoms were recorded, such as purpura, gastrointestinal bleeding, arthralgia or arthritis, and renal damage. All Standard tests including routine blood and urine tests, C-reactive protein, D-dimer, and cystatin C were determined. Continuous variables were described as mean ± SD and compared by the independent sample *t-test.* Categorical variables were recorded as percentages and analyzed using the Chi-square test.

### Serum metabonomics analysis based on UPLC-Q-TOF-MS/MS

Detailed information about sample collection, sample preparation, settings of chromatographic separation and MS determination, data preprocessing and data pretreatment can be found in Supplementary Materials.

### Metabolic data analysis

The unsupervised principle component analysis (PCA) was employed to obtain a general separation of all samples. Subsequently, multivariate predictive modeling by the supervised partial least-squares discriminant analysis (PLS-DA) was applied to reveal the differences in metabolic profiling between groups, and the correspond variable importance in the projection (VIP) was calculated as well. To validate and avoid the overfitting problem of PLS-DA model, a seven fold cross-validation was used to optimize the number of components in the model. 200 times of permutation tests were also performed: constructed 200 models with randomly assigned labels for the samples, then compared the goodness of fit (parameters Q2 and R2) of the original model with the goodness of fit of permuted models. Finally, an ANOVA of the cross-validated residuals (CV-ANOVA) test was executed as a significance test, to verify the validity of PLS-DA models. Only if the permutation test and the CV-ANOVA test provided satisfactory results simultaneously, the PLS-DA model was regarded as a valid one. The independent sample *t-test* was performed to determine the significance of each metabolite. A potential biomarker was extracted if the VIP-value was more than 1.0 while the P value of the *t-test* was less than 0.05.

To evaluate the validity of the metabolic biomarkers for detecting children with HSP and HSPN, the receiver operating characteristic curve (ROC) analyses were employed. Top biomarkers with better sensitivity and specificity were selected and combined with the clinical risk factors for renal involvement in HSP using multivariate logistic regression, to enhance the diagnostic capability for HSPN.

The permutation test and the CV-ANOVA test of PLS-DA models were carried out on SIMCA-P (version 11.5; Umetrics, Malmo, Sweden). Other data analysis was performed on R platform. All statistical tests were two-sided, and the significant level was set at P < 0.05.

### Identification of potential biomarkers

The identification of potential biomarkers was based on the retention time, the mass assignment and the MS/MS ion fragments, by comparing with information of web database and MassFragment application manager (Waters MassLynx version4.1, Waters). Mass tolerance between the measured m/z and the actual value was set to within 20 ppm. Online information of biomarkers was acquired from databases HMDB, METLIN, ChemSpider and KEGG.

## SUPPLEMENTARY MATERIALS FIGURES AND TABLES





## Abbreviations

AUC: Area under the curve
CKD: Chronic kidney disease
CV-ANOVA: ANOVA of the cross-validated residuals
DPA: Docosapentaenoic acid
DHA: Docosahexaenoic acid
DGLA: 8,11,14-Eicosatrienoic acid
ESI: Electrospray ionization
EPA: Eicosapentaenoic acid
GLA: Gamma-linolenic acid
HC: Healthy children
HSP: Henoch-Schonlein purpura
HSPN: Henoch-Schonlein purpura nephritis
HSPWN: Henoch-Schonlein purpura without nephritis
IgAN: IgA nephropathy
PAI-1: Plasminogen activator inhibitor type-1
PCA: Principle component analysis
PCS: p-Cresol sulfate
PGE1: Prostaglandin E1
PLS-DA: Partial least-squares discriminant analysis
PPAR-γ: Peroxisomal proliferator-activated receptor- γ
QC: Quality control
ROC: Receiver operating characteristic curve
RT: Retention time
UA: Urinanalysis
UPLC-Q-TOF-MS/MS: Ultra perfomance liquid chromatography quadrupole time-of-flight tandem mass spectrometry
VIP: variable importance in the projection
3-HIBA: (S)-3-hydroxyisobutyric acid
5-propyl FPA: 3-carboxy-4-methyl-5-pentyl-2-furanpropanoic acid
