# Supplementary material for: Biomarkers identification by a combined clinical and metabonomics analysis in Henoch-Schonlein purpura nephritis children
Source: Oncotarget. 2017 Nov 24;8(69):114239–50. doi: 10.18632/oncotarget.23207 (PMC5768399; doi:10.18632/oncotarget.23207)
Supplement: Supplementary file 1 [file oncotarget-08-114239-s001.pdf]

Biomarkers identification by a combined clinical and metabonomics analysis in Henoch-Schonlein purpura nephritis children

SUPPLEMENTARY MATERIALS

Sample collection

A 5 ml of whole blood was collected from each participant into a vacuum blood collection tube containing / which contained anticoagulant dipotassium ethylene diamine tetraacetic acid (EDTA). Fresh blood was centrifuged at 4000rpm for 10 min, then the supernatant was diluted and frozen at -80°C until / for further analysis.

Sample preparation

The data quality and instrument performance were monitored using quality control (QC) samples prepared by pooled mixing equal volumes of different individual serum samples. One QC sample was run for every 15 serum samples. After the serum and QC samples were thawed in a 4 °C refrigerator, a volume of 350μl of each serum sample was mixed with two volumes (700μl) of methanol. The mixture was vortex-mixed for 2 min and incubated at room temperature for 10 min, followed by centrifuged at 12000 rpm for 12 min at 4°C.

The supernatant was transferred into a clear tube and evaporated to dryness under nitrogen. The residue was redissolved in 350μl of a mixture of acetonitrile and water (1:2), vortex-mixed for 2 min, then centrifuged at 12000 rpm for 15 min at 4°C. The supernatant was then placed into the sample vial for UPLC-Q-TOF-MSMS analysis. One blank sample (100% acetonitrile) was run for every 5 serum samples.

Chromatographic separation and MS

A 2ul aliquot of the sample solution was injected into an Acquity UPLC^{TM} BEH C_{18} column (100mm 2.1 mm; id. 1.7um; Waters Corp.). The flow rate of the mobile phase was 350ul/min. Analytes were elution from the column under a gradient (solvent A, 0.1% formic acid in water, and solvent B, acetonotrile). A liner elution gradient was used as follows: 2%B for 0.5 min, 2-20%B over 0.5-2.0 min, 20-70%B over 2.0-6.0 min, 70-100%B in a further 4 min, and 100% maintained 2min (10-12min), followed by re-equilibration to the initial conditions in 6min (12-18min). The serum samples were injected alternately between three groups. Q-TOF MSMS was performed with a mass spectrometer (Micromass Q-TOF mass spectrometer; Waters Corp.) equipped with an electrospray ionization (ESI) interface operated in the negative ion mode (ESI-). The scan mass ranged from 50 to 1000 m/z. The capillary voltage and sample cone voltage were set at 3000V and 35V, respectively. The desolvation gas (nitrogen) flow was set to 600L/h at a temperature of 320°C. The cone gas (nitrogen) were set to 50L/h and the source temperature was 110°C. The collision gas was argon and the collision energy was set at 6eV. The MCP detector voltage was set at 2550V. The data acquisition rate was set to 0.4s, with a 0.1s interscan delay. The Q-TOF MS/MS data were collected in centroid mode. All sample analyses were acquired by the lock spray in order to ensure good accuracy and repeatability. A concentration of 200pg/ml leucine-enkephalin calibrator was used as lock mass (m/z 554.2615) in ESI-.

Data preprocessing and pretreatment

After UPLC-MS/MS determination, the raw metabolic data was imported into MakerLynx software. Peak detection and arrangement were performed using MakerLynx ApexTrack peak integration. Subsequently, a matrix for multivariate analysis was generated by the software, including the information of retention time (RT), mass to charge ratio (m/z), and respective ion intensities. The intensity of each ion was normalized to the total peak intensities in the relative chromatogram, aimed at reducing the systemic variation during the whole experiment. If ion intensity in the corresponding sample was less than the detection limit, it was assigned a value of zero. A data filtering procedure was handled based on the '80 rule' [1] to remove the features with overmuch missing values. To reduce the impact of differences of magnitude, data scaled to Pareto before multivariate statistical analysis.

REFERENCE

1. Bijlsma S, Bobeldijk I, Verheij ER, Ramaker R, Kochhar S, Macdonald IA, van Ommen B, Smilde AK. Large-scale human metabolomics studies: a strategy for data (pre-) processing and validation. Anal Chem. 2006; 78: 567-574.

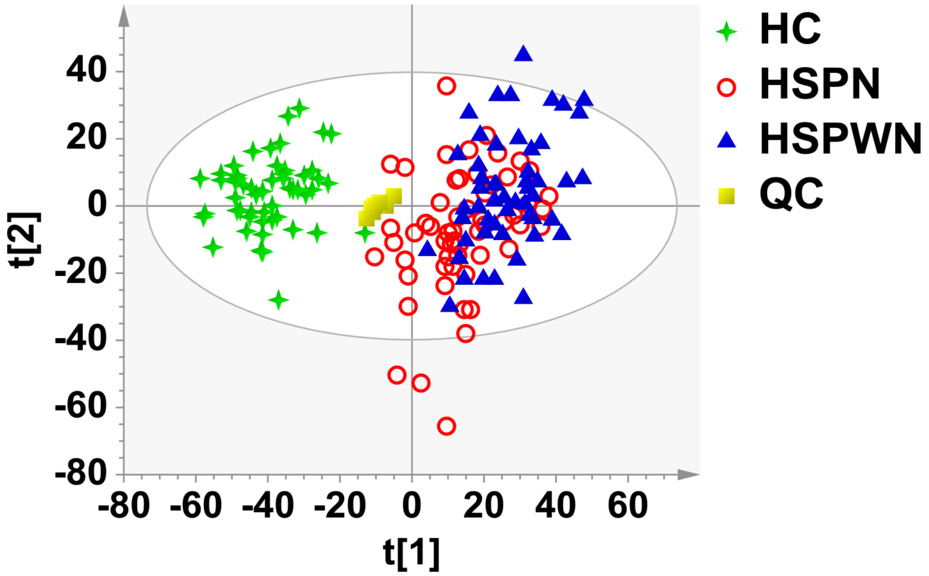

Supplementary Figure 1: PCA scores plot of all injections.

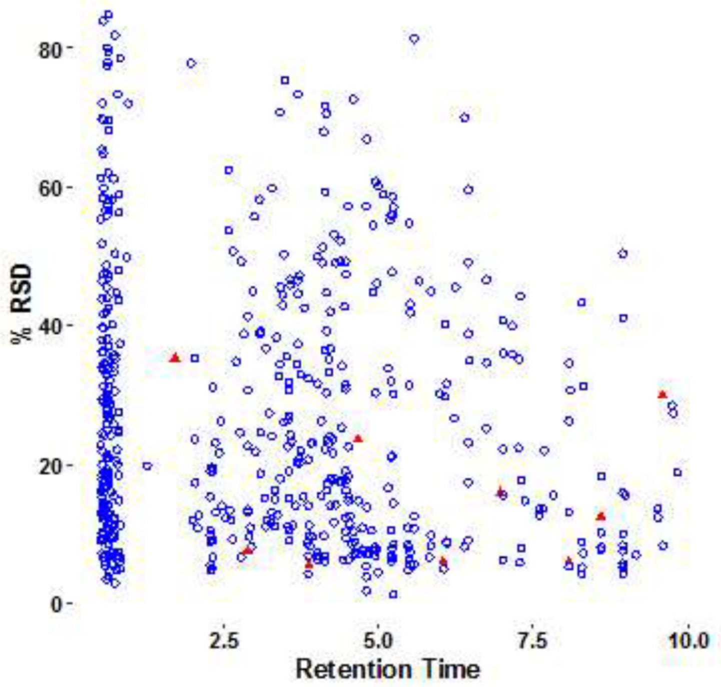

Supplementary Figure 2: Scatter plot of the %RSD values of all metabolites from QCsamples. The red triangles represent 9 selected metabolites.

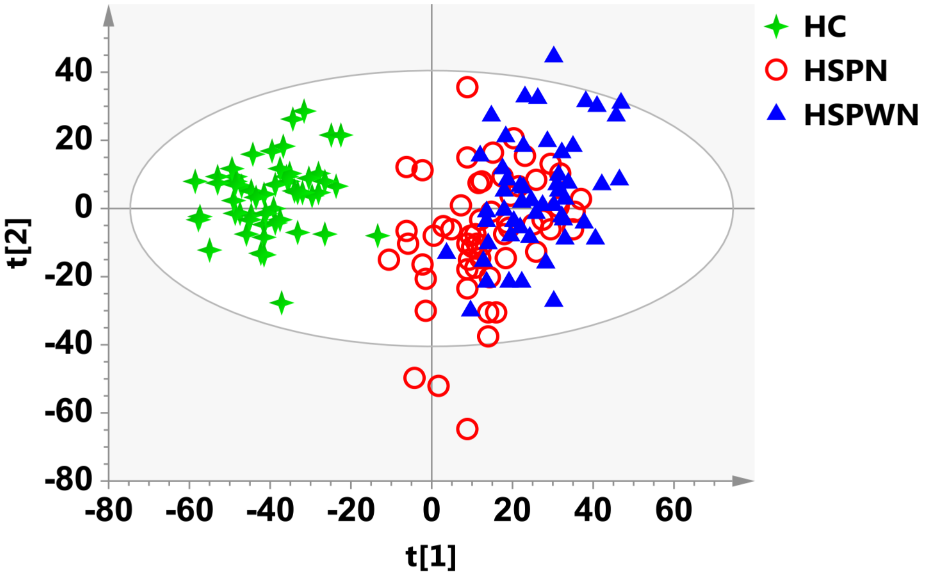

Supplementary Figure 3: PCA scores plot of HSPN, HSPWN and HC.

Supplementary Table 1: CV-ANOVA test of PLS-DA model of HSP and HC groups

HSP vs. HC	SS	DF	MS	F	P	SD
Total corr.	161	161	1.00			1.00
Regression	149.66	6	24.94	340.89	0.000	4.99
Residual	11.34	155	0.07			0.27

Supplementary Table 2: CV-ANOVA test of PLS-DA model of HSPWN and HC groups

HSPWN vs. HC	SS	DF	MS	F	P	SD
Total corr.	104	104	1.00			1.00
Regression	97.11	4	24.28	352.36	0.000	4.93
Residual	6.89	100	0.07			0.26

Supplementary Table 3: CV-ANOVA test of PLS-DA model of HSPN and HC groups

HSPN vs. HC	SS	DF	MS	F	P	SD
Total corr.	109.00	109	1.00			1.00
Regression	101.54	4	25.38	357.14	0.000	5.04
Residual	7.46	105	0.07			0.27

Supplementary Table 4: CV-ANOVA test of PLS-DA model of HSPN and HSPWN groups

HSPN vs. HSPWN	SS	DF	MS	F	P	SD
Total corr.	108.00	108	1			1.00
Regression	54.838	2	27.42	54.67	0.000	5.24
Residual	53.162	106	0.50			0.71

Supplementary Data: Detailed information of identification of 7 biomarkers.

See Supplementary File 1
