# Supplementary material for: Biomarkers identification by a combined clinical and metabonomics analysis in Henoch-Schonlein purpura nephritis children
Source: Oncotarget. 2017 Nov 24;8(69):114239–50. doi: 10.18632/oncotarget.23207 (PMC5768399; doi:10.18632/oncotarget.23207)

**Supplementary Data**

1. **(S)-3-Hydroxyisobutyric acid**


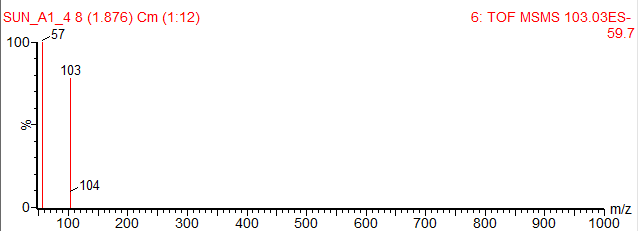


**LC-MS/MS Spectrum - Quattro_QQQ 25V, Negative (Annotated)**

**
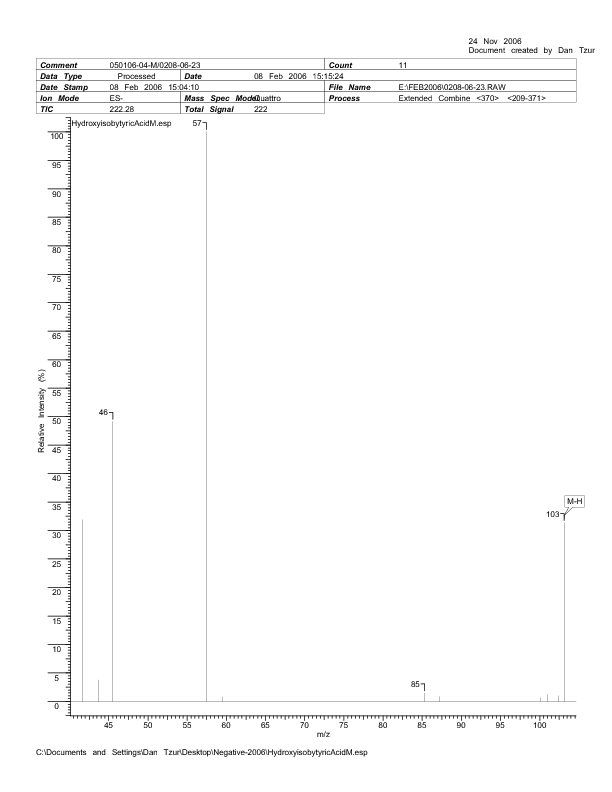
**

Input:

| 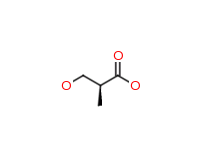 | **ID (job)** | 2 |
| --- | --- | --- |
|  | **Mass (Da)** | 104.0473 |
|  | **Formula** | C_4_H_8_O_3_ |
|  | **DBE** | 2 |

Experiment:

| **Product ion(s) (Da)** | 103.0402 104.0472 57.0332 +/- 0.01 in negative mode, structure filter off |
| --- | --- |
| **DBE** | 0 to 50 |
| **Electron count** | both |
| **Maximum H deficit** | 6 |
| **Fragment number of bonds** | 4 |
| **Scoring** | aromatic: 6, multiple: 4, ring: 2, phenyl: 8, other: 1 H-deficit: 0, hetero modifier: 0.5, max score: 16 |
| **Order:** | mass |
| **Plot:** | show hide  |

Results:

| \| **57.0332** \| ¬- (+0H) \| \| --- \| --- \| \| 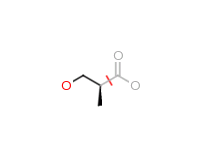 \| \| \| 57.0340 (-0.8.mDa) (S:1.0, B:1) C_3_H_5_O (-CH_2_O_2_) \| \| |
| --- | --- | --- | --- | --- | --- | --- |


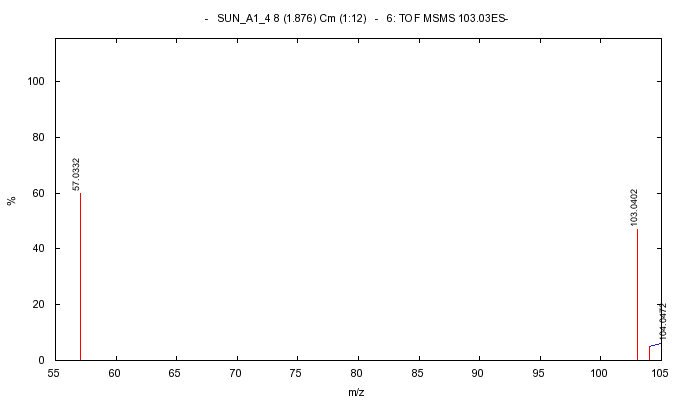


1. **p-Cresol sulfate (PCS)**

Input:

| 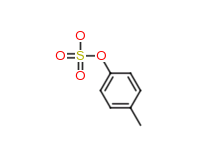 | **ID (job)** | 96 |
| --- | --- | --- |
|  | **Mass (Da)** | 188.0143 |
|  | **Formula** | C_7_H_8_O_4_S |
|  | **DBE** | 5 |

Experiment:

| **Product ion(s) (Da)** | 107.0529 108.0514 125.0959 141.8502 142.9429 152.9054 186.1790 187.0066 187.9985 189.0087 196.7021 79.9579 80.9662 +/- 0.01 in negative mode, structure filter off |
| --- | --- |
| **DBE** | 0 to 50 |
| **Electron count** | both |
| **Maximum H deficit** | 6 |
| **Fragment number of bonds** | 4 |
| **Scoring** | aromatic: 6, multiple: 4, ring: 2, phenyl: 8, other: 1 H-deficit: 0, hetero modifier: 0.5, max score: 16 |
| **Order:** | mass |
| **Plot:** | show hide  |

Results:

| \| **107.0529** \| ¬- (+2H) \| \| --- \| --- \| \| 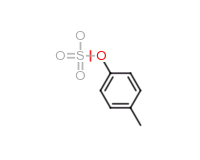 \| \| \| 107.0497 (+3.2.mDa) (S:1.0, B:1) C_7_H_7_O (-O_3_S) \| \| | \| **80.9662** \| ¬- (+2H) \| \| --- \| --- \| \| 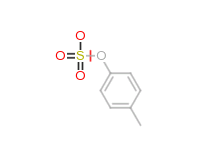 \| \| \| 80.9646 (+1.6.mDa) (S:1.0, B:1) HO_3_S (-C_7_H_6_O) \| \| |
| --- | --- | --- | --- | --- | --- | --- | --- | --- | --- | --- | --- | --- | --- |


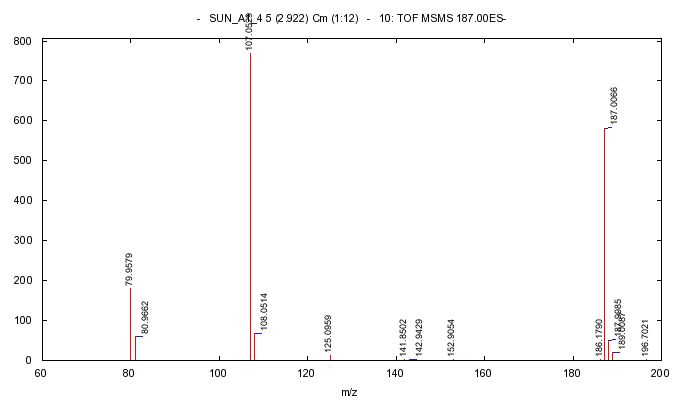


1. **Alpha-dimorphecolic acid (9(S)-HODE)**

**
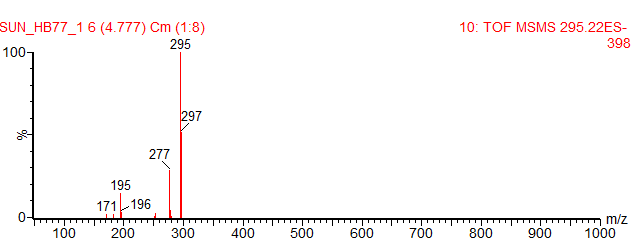
**


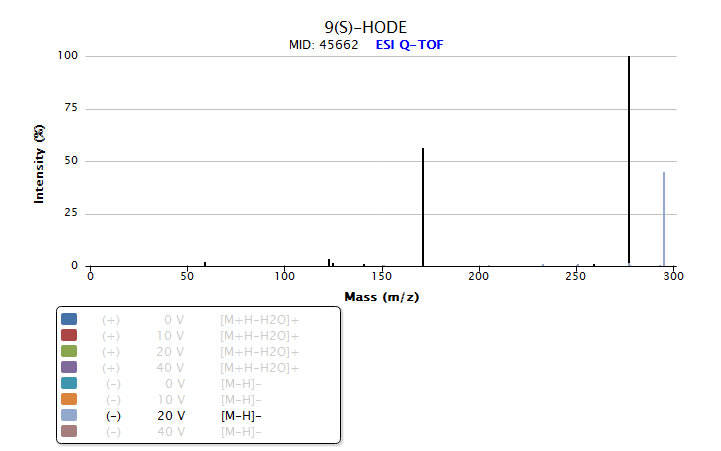


Input:

| 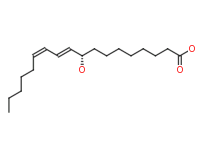 | **ID (job)** | 29 |
| --- | --- | --- |
|  | **Mass (Da)** | 296.2351 |
|  | **Formula** | C_18_H_32_O_3_ |
|  | **DBE** | 4 |

Experiment:

| **Product ion(s) (Da)** | 171.1079 183.1316 195.1375 196.1463 251.2272 253.2169 277.2215 278.2239 279.2512 295.2277 296.2283 297.1674 +/- 0.01 in negative mode, structure filter off |
| --- | --- |
| **DBE** | 0 to 50 |
| **Electron count** | both |
| **Maximum H deficit** | 6 |
| **Fragment number of bonds** | 4 |
| **Scoring** | aromatic: 6, multiple: 4, ring: 2, phenyl: 8, other: 1 H-deficit: 0, hetero modifier: 0.5, max score: 16 |
| **Order:** | mass |
| **Plot:** | show hide  |

Results:

| \| **277.2215** \| ¬- (+0H) \| \| --- \| --- \| \| 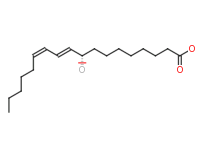 \| \| \| 277.2168 (+4.7.mDa) (S:0.5, B:1) C_18_H_29_O_2_ (-H_2_O) \| \| | \| **277.2215** \| ¬- (+0H) \| \| --- \| --- \| \| 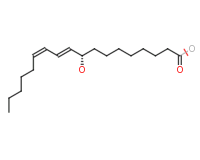 \| \| \| 277.2168 (+4.7.mDa) (S:0.5, B:1) C_18_H_29_O_2_ (-H_2_O) \| \| | \| **253.2169** \| ¬- (+5H) \| \| --- \| --- \| \| 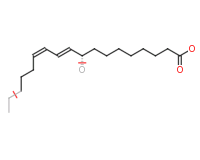 \| \| \| 253.2168 (+0.1.mDa) (S:1.5, B:2) C_16_H_29_O_2_ (-C_2_H_2_O) \| \| |
| --- | --- | --- | --- | --- | --- | --- | --- | --- | --- | --- | --- | --- | --- | --- | --- | --- | --- | --- | --- | --- |
| \| **253.2169** \| ¬- (+5H) \| \| --- \| --- \| \| 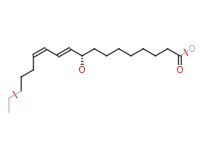 \| \| \| 253.2168 (+0.1.mDa) (S:1.5, B:2) C_16_H_29_O_2_ (-C_2_H_2_O) \| \| | \| **253.2169** \| ¬- (+4H) \| \| --- \| --- \| \| 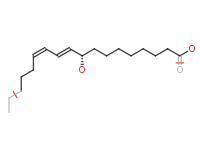 \| \| \| 253.2168 (+0.1.mDa) (S:3.0, B:2) C_16_H_29_O_2_ (-C_2_H_2_O) \| \| | \| **196.1463** \| ¬^.^- (+3H) \| \| --- \| --- \| \| 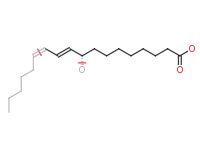 \| \| \| 196.1463 (-0.0.mDa) (S:4.5, B:2) C_12_H_20_O_2_ (-C_6_H_11_O) \| \| |
| \| **196.1463** \| ¬^.^- (+3H) \| \| --- \| --- \| \| 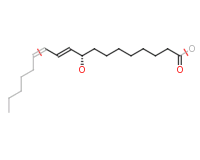 \| \| \| 196.1463 (-0.0.mDa) (S:4.5, B:2) C_12_H_20_O_2_ (-C_6_H_11_O) \| \| | \| **196.1463** \| ¬^.^- (+2H) \| \| --- \| --- \| \| 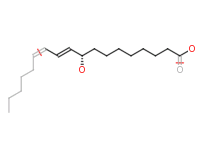 \| \| \| 196.1463 (-0.0.mDa) (S:6.0, B:2) C_12_H_20_O_2_ (-C_6_H_11_O) \| \| | \| **183.1316** \| ¬- (+3H) \| \| --- \| --- \| \| 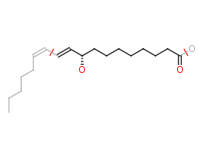 \| \| \| 183.1385 (-6.9.mDa) (S:1.5, B:2) C_11_H_19_O_2_ (-C_7_H_12_O) \| \| |
| \| **183.1316** \| ¬- (+3H) \| \| --- \| --- \| \| 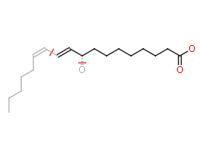 \| \| \| 183.1385 (-6.9.mDa) (S:1.5, B:2) C_11_H_19_O_2_ (-C_7_H_12_O) \| \| | \| **183.1316** \| ¬- (+2H) \| \| --- \| --- \| \| 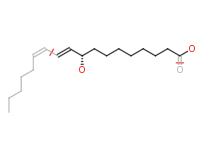 \| \| \| 183.1385 (-6.9.mDa) (S:3.0, B:2) C_11_H_19_O_2_ (-C_7_H_12_O) \| \| | \| **171.1079** \| ¬- (+0H) \| \| --- \| --- \| \| 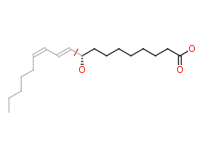 \| \| \| 171.1021 (+5.8.mDa) (S:1.0, B:1) C_9_H_15_O_3_ (-C_9_H_16_) \| \| |
|  |  |  |


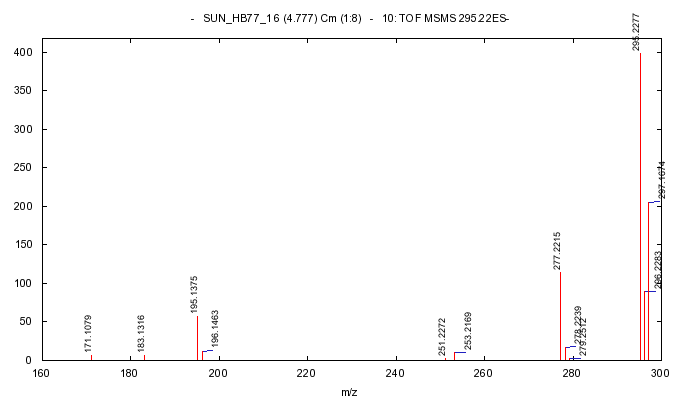


1. **3-carboxy-4-methyl-5-pentyl-2-furanpropanoic acid (5-propyl FPA)**

**
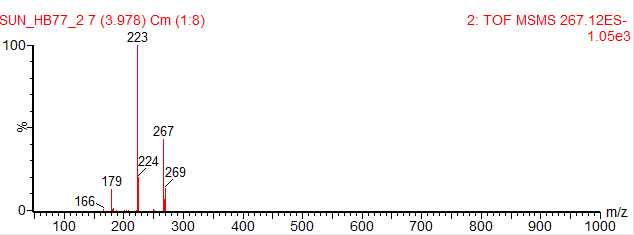
**

Input:

| 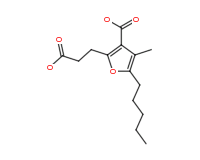 | **ID (job)** | 85 |
| --- | --- | --- |
|  | **Mass (Da)** | 268.1311 |
|  | **Formula** | C_14_H_20_O_5_ |
|  | **DBE** | 6 |

Experiment:

| **Product ion(s) (Da)** | 166.0940 179.1420 180.1462 183.1108 186.9160 201.1401 205.1171 207.1830 221.7319 223.1312 224.1355 225.1186 249.1274 251.1900 266.1054 267.1184 268.1361 269.1581 +/- 0.01 in negative mode, structure filter off |
| --- | --- |
| **DBE** | 0 to 50 |
| **Electron count** | both |
| **Maximum H deficit** | 6 |
| **Fragment number of bonds** | 4 |
| **Scoring** | aromatic: 6, multiple: 4, ring: 2, phenyl: 8, other: 1 H-deficit: 0, hetero modifier: 0.5, max score: 16 |
| **Order:** | mass |
| **Plot:** | show hide  |

Results:

| \| **267.1184** \| ¬- (+1H) \| \| --- \| --- \| \| 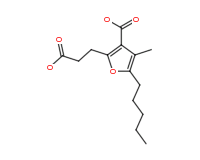 \| \| \| 267.1232 (-4.9.mDa) (S:3.0, B:0) C_14_H_19_O_5_ (-none) \| \| | \| **223.1312** \| ¬- (+2H) \| \| --- \| --- \| \| 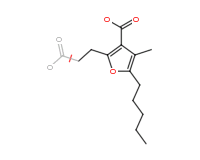 \| \| \| 223.1334 (-2.2.mDa) (S:1.0, B:1) C_13_H_19_O_3_ (-CO_2_) \| \| | \| **223.1312** \| ¬- (+2H) \| \| --- \| --- \| \| 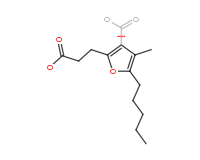 \| \| \| 223.1334 (-2.2.mDa) (S:1.0, B:1) C_13_H_19_O_3_ (-CO_2_) \| \| |
| --- | --- | --- | --- | --- | --- | --- | --- | --- | --- | --- | --- | --- | --- | --- | --- | --- | --- | --- | --- | --- |
| \| **179.1420** \| ¬- (+3H) \| \| --- \| --- \| \| 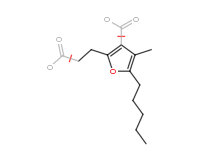 \| \| \| 179.1436 (-1.6.mDa) (S:2.0, B:2) C_12_H_19_O (-C_2_O_4_) \| \| |  |  |


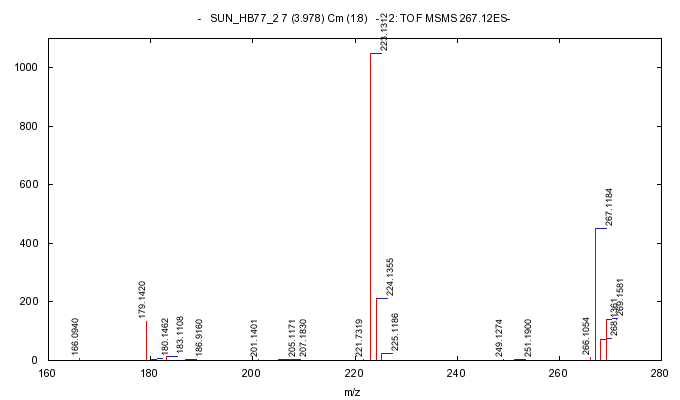


1. **LysoPC(15:0)**


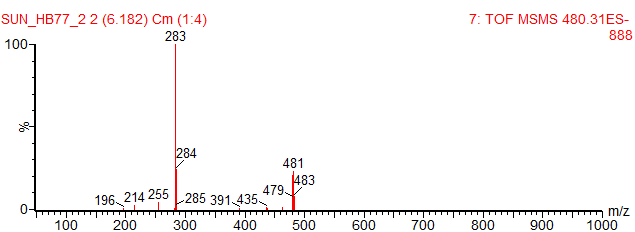


Input:

| 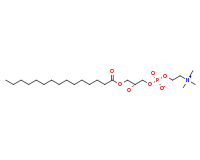 | **ID (job)** | 52 |
| --- | --- | --- |
|  | **Mass (Da)** | 481.3168 |
|  | **Formula** | C_23_H_48_NO_7_P |
|  | **DBE** | 2 |

Experiment:

| **Product ion(s) (Da)** | 196.0296 214.0464 255.2308 282.2803 283.2658 284.2711 285.2776 391.2698 435.3584 436.3726 437.3678 438.4088 463.3585 479.3558 480.3234 481.3467 482.3503 483.3471 +/- 0.01 in negative mode, structure filter off |
| --- | --- |
| **DBE** | 0 to 50 |
| **Electron count** | both |
| **Maximum H deficit** | 6 |
| **Fragment number of bonds** | 4 |
| **Scoring** | aromatic: 6, multiple: 4, ring: 2, phenyl: 8, other: 1 H-deficit: 0, hetero modifier: 0.5, max score: 16 |
| **Order:** | mass |
| **Plot:** | show hide  |

Results:

| \| **283.2658** \| ¬- (+3H) \| \| --- \| --- \| \| 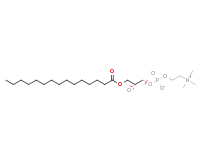 \| \| \| 283.2637 (+2.1.mDa) (S:1.0, B:2) C_18_H_35_O_2_ (-C_5_H_12_NO_5_P) \| \| | \| **283.2658** \| ¬- (+2H) \| \| --- \| --- \| \| 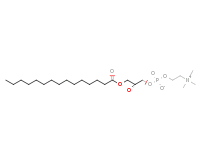 \| \| \| 283.2637 (+2.1.mDa) (S:2.5, B:2) C_18_H_35_O_2_ (-C_5_H_12_NO_5_P) \| \| | \| **255.2308** \| ¬- (+2H) \| \| --- \| --- \| \| 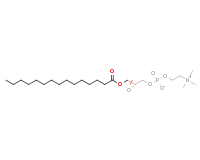 \| \| \| 255.2324 (-1.6.mDa) (S:1.0, B:1) C_16_H_31_O_2_ (-C_7_H_16_NO_5_P) \| \| |
| --- | --- | --- | --- | --- | --- | --- | --- | --- | --- | --- | --- | --- | --- | --- | --- | --- | --- | --- | --- | --- |
| \| **196.0296** \| ¬- (+2H) \| \| --- \| --- \| \| 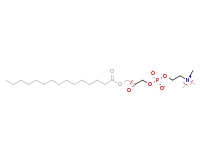 \| \| \| 196.0375 (-7.9.mDa) (S:2.0, B:3) C_5_H_11_NO_5_P (-C_18_H_36_O_2_) \| \| | \| **196.0296** \| ¬- (+2H) \| \| --- \| --- \| \| 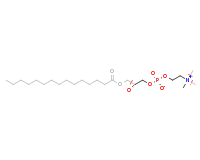 \| \| \| 196.0375 (-7.9.mDa) (S:2.0, B:3) C_5_H_11_NO_5_P (-C_18_H_36_O_2_) \| \| | \| **196.0296** \| ¬- (+2H) \| \| --- \| --- \| \| 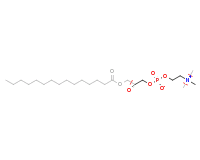 \| \| \| 196.0375 (-7.9.mDa) (S:2.0, B:3) C_5_H_11_NO_5_P (-C_18_H_36_O_2_) \| \| |
|  |  |  |


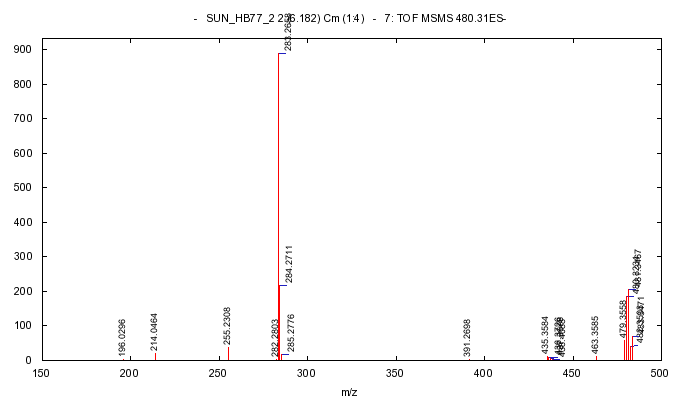


1. **LysoSM(d18:1)**


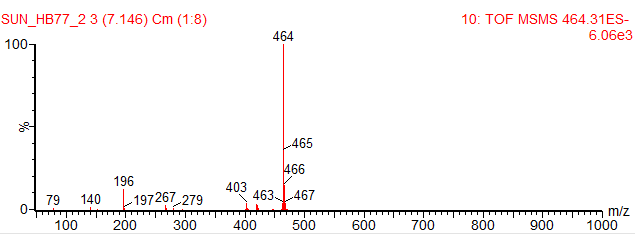


Input:

| 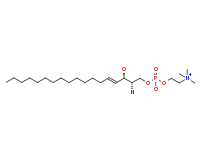 | **ID (job)** | 48 |
| --- | --- | --- |
|  | **Mass (Da)** | 465.3457 |
|  | **Formula** | C_23_H_50_N_2_O_5_P |
|  | **DBE** | 1.5 |

Experiment:

| **Product ion(s) (Da)** | 140.0082 141.0156 152.9960 196.0365 197.0117 267.2692 268.2733 279.2271 280.2360 295.2843 401.3541 402.0677 403.2678 404.2562 405.2865 406.3046 419.3540 420.3629 421.3662 422.3609 423.3129 445.3435 447.3656 461.7406 462.3829 463.3414 464.3149 465.3225 466.3246 467.3343 468.4710 469.4083 470.7247 471.6063 78.9563 +/- 0.01 in negative mode, structure filter off |
| --- | --- |
| **DBE** | 0 to 50 |
| **Electron count** | both |
| **Maximum H deficit** | 6 |
| **Fragment number of bonds** | 4 |
| **Scoring** | aromatic: 6, multiple: 4, ring: 2, phenyl: 8, other: 1 H-deficit: 0, hetero modifier: 0.5, max score: 16 |
| **Order:** | mass |
| **Plot:** | show hide  |

Results:

| \| **423.3129** \| ¬^.^- (+5H) \| \| --- \| --- \| \| 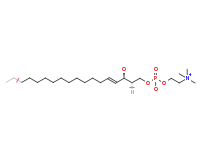 \| \| \| 423.3114 (+1.5.mDa) (S:1.5, B:2) C_21_H_46_NO_5_P (-C_2_H_3_N) \| \| | \| **406.3046** \| ¬- (+5H) \| \| --- \| --- \| \| 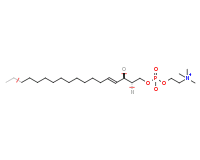 \| \| \| 406.3086 (-4.0.mDa) (S:2.0, B:3) C_21_H_45_NO_4_P (-C_2_H_4_NO) \| \| | \| **406.3046** \| ¬^.^- (+3H) \| \| --- \| --- \| \| 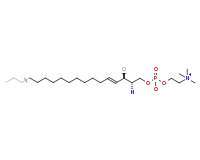 \| \| \| 406.2960 (+8.6.mDa) (S:1.5, B:2) C_20_H_43_N_2_O_4_P (-C_3_H_6_O) \| \| |
| --- | --- | --- | --- | --- | --- | --- | --- | --- | --- | --- | --- | --- | --- | --- | --- | --- | --- | --- | --- | --- |
| \| **405.2865** \| ¬- (+2H) \| \| --- \| --- \| \| 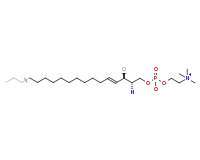 \| \| \| 405.2882 (-1.7.mDa) (S:1.5, B:2) C_20_H_42_N_2_O_4_P (-C_3_H_7_O) \| \| | \| **403.2678** \| ¬- (+0H) \| \| --- \| --- \| \| 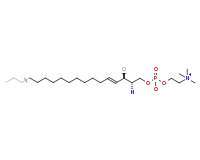 \| \| \| 403.2726 (-4.8.mDa) (S:1.5, B:2) C_20_H_40_N_2_O_4_P (-C_3_H_9_O) \| \| | \| **403.2678** \| ¬- (+0H) \| \| --- \| --- \| \| 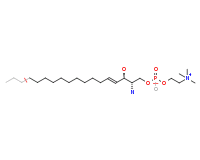 \| \| \| 403.2726 (-4.8.mDa) (S:2.0, B:2) C_20_H_40_N_2_O_4_P (-C_3_H_9_O) \| \| |
| \| **280.2360** \| ¬^.^- (+0H) \| \| --- \| --- \| \| 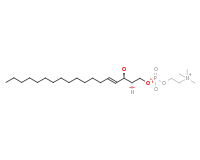 \| \| \| 280.2402 (-4.2.mDa) (S:1.5, B:2) C_18_H_32_O_2_ (-C_5_H_17_N_2_O_3_P) \| \| | \| **280.2360** \| ¬- (-1H) \| \| --- \| --- \| \| 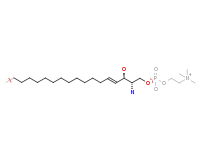 \| \| \| 280.2277 (+8.3.mDa) (S:2.0, B:2) C_17_H_30_NO_2_ (-C_6_H_19_NO_3_P) \| \| | \| **196.0365** \| ¬- (+3H) \| \| --- \| --- \| \| 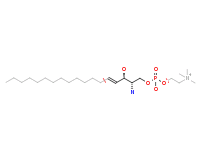 \| \| \| 196.0375 (-1.0.mDa) (S:1.5, B:2) C_5_H_11_NO_5_P (-C_18_H_38_N) \| \| |
| \| **196.0365** \| ¬- (+1H) \| \| --- \| --- \| \| 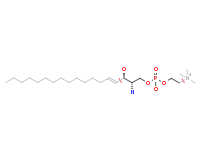 \| \| \| 196.0375 (-1.0.mDa) (S:1.5, B:2) C_5_H_11_NO_5_P (-C_18_H_38_N) \| \| | \| **152.9960** \| ¬- (+2H) \| \| --- \| --- \| \| 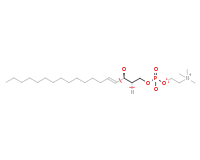 \| \| \| 152.9953 (+0.7.mDa) (S:2.0, B:3) C_3_H_6_O_5_P (-C_20_H_43_N_2_) \| \| | \| **141.0156** \| ¬^.^- (+4H) \| \| --- \| --- \| \| 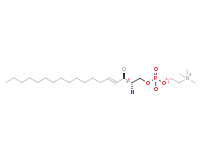 \| \| \| 141.0191 (-3.5.mDa) (S:1.5, B:2) C_2_H_8_NO_4_P (-C_21_H_41_NO) \| \| |
| \| **140.0082** \| ¬- (+3H) \| \| --- \| --- \| \| 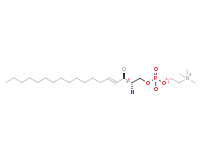 \| \| \| 140.0113 (-3.1.mDa) (S:1.5, B:2) C_2_H_7_NO_4_P (-C_21_H_42_NO) \| \| | \| **78.9563** \| ¬- (+1H) \| \| --- \| --- \| \| 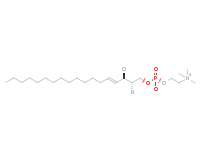 \| \| \| 78.9585 (-2.2.mDa) (S:1.5, B:2) O_3_P (-C_23_H_49_N_2_O_2_) \| \| | \| **78.9563** \| ¬- (+1H) \| \| --- \| --- \| \| 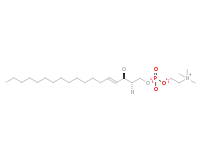 \| \| \| 78.9585 (-2.2.mDa) (S:1.5, B:2) O_3_P (-C_23_H_49_N_2_O_2_) \| \| |
| \| **78.9563** \| ¬- (+2H) \| \| --- \| --- \| \| 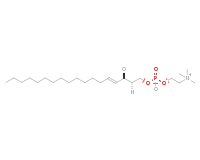 \| \| \| 78.9585 (-2.2.mDa) (S:2.0, B:3) O_3_P (-C_23_H_49_N_2_O_2_) \| \| | \| **78.9563** \| ¬- (+1H) \| \| --- \| --- \| \| 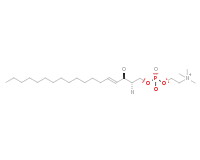 \| \| \| 78.9585 (-2.2.mDa) (S:5.0, B:3) O_3_P (-C_23_H_49_N_2_O_2_) \| \| |  |


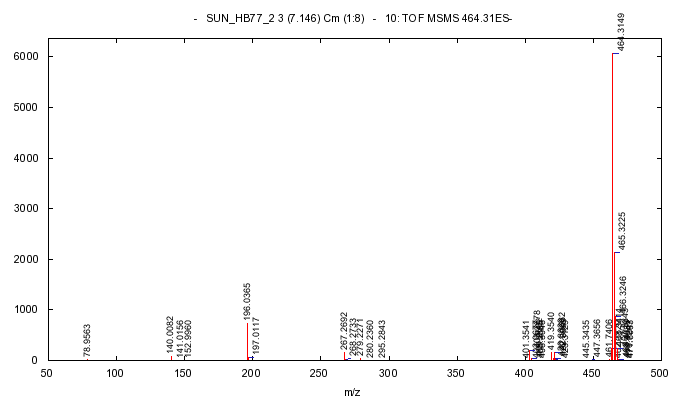


**7. 8,11,14-Eicosatrienoic acid (DGLA)**

**
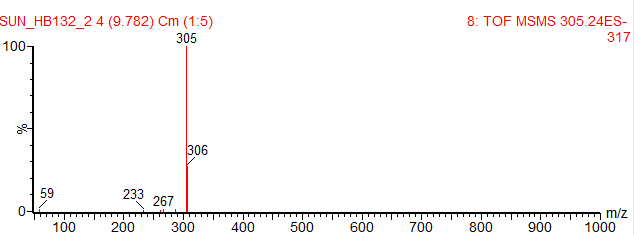
**

Input:

| 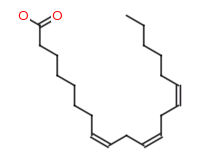 | **ID (job)** | 37 |
| --- | --- | --- |
|  | **Mass (Da)** | 306.2559 |
|  | **Formula** | C_20_H_34_O_2_ |
|  | **DBE** | 5 |

Experiment:

| **Product ion(s) (Da)** | 233.2092 261.2608 267.2155 287.2439 305.2488 306.2563 59.0133 +/- 0.01 in negative mode, structure filter off |
| --- | --- |
| **DBE** | 0 to 50 |
| **Electron count** | both |
| **Maximum H deficit** | 6 |
| **Fragment number of bonds** | 4 |
| **Scoring** | aromatic: 6, multiple: 4, ring: 2, phenyl: 8, other: 1 H-deficit: 0, hetero modifier: 0.5, max score: 16 |
| **Order:** | mass |
| **Plot:** | show hide  |

Results:

| \| **287.2439** \| ¬- (+0H) \| \| --- \| --- \| \| 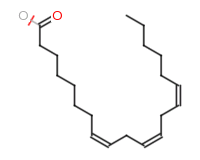 \| \| \| 287.2375 (+6.4.mDa) (S:0.5, B:1) C_20_H_31_O (-H_2_O) \| \| | \| **261.2608** \| ¬- (+2H) \| \| --- \| --- \| \| 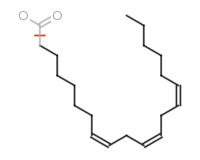 \| \| \| 261.2582 (+2.6.mDa) (S:1.0, B:1) C_19_H_33_ (-CO_2_) \| \| | \| **261.2608** \| ¬- (+5H) \| \| --- \| --- \| \| 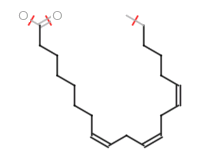 \| \| \| 261.2582 (+2.6.mDa) (S:3.5, B:3) C_19_H_33_ (-CO_2_) \| \| |
| --- | --- | --- | --- | --- | --- | --- | --- | --- | --- | --- | --- | --- | --- | --- | --- | --- | --- | --- | --- | --- |
| \| **59.0133** \| ¬- (+2H) \| \| --- \| --- \| \| 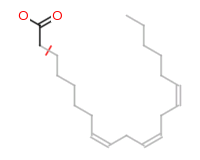 \| \| \| 59.0133 (-0.0.mDa) (S:1.0, B:1) C_2_H_3_O_2_ (-C_18_H_30_) \| \| |  |  |


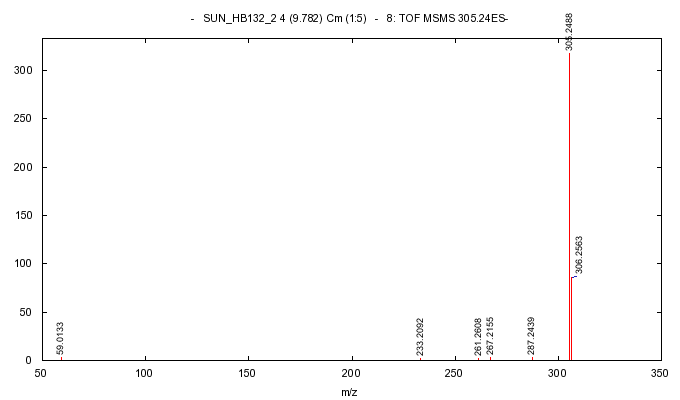

Supplement: Supplementary file 2 [file oncotarget-08-114239-s002.docx]
